# A new set of metrics and framework to assess the colonization potential of riverscapes by wind-dispersed plant species

**DOI:** 10.1038/s41598-023-47477-y

**Published:** 2023-11-16

**Authors:** Thomas C. Wagner, Romy Woellner

**Affiliations:** Restoration Ecology, Technische Universität München, Emil-Ramann-Str. 6, 95354 Freising, Germany

**Keywords:** Conservation biology, Population dynamics, Restoration ecology, Riparian ecology

## Abstract

Quantifying the potential of a braided riverscape to be colonized by a plant species is essential for assessing the ecological state of the river and provides an important basis for nature conservation planning and the implementation of restoration measures. Common connectivity indices are largely unsuitable for describing the situation for the mostly wind-dispersed plant species. Our approach provides a set of comparable metrics that allows the quantification of the colonization potential of riverscapes at the patch and riverscape level. We propose a set of cell-based, spatially explicit measures that can easily be implemented. We demonstrate their application using two typical plant species and three riverscapes with different habitat configurations as examples. Our metrics consider shape, size and the spatial configuration of habitat patches, along with the dispersal characteristics of the respective species. The metrics provide a linear, balanced, and realistic representation of the colonization potential at the cell, patch, and riverscape levels. The results are comparable between different riverscapes and species, can be easily extended and used for further modeling. The metrics provide a valuable tool for the planning and evaluation of conservation, restoration, and reintroduction measures and close the gap between habitat availability analyses and large-scale terrestrial connectivity indices.

## Introduction

**Figure 1:**
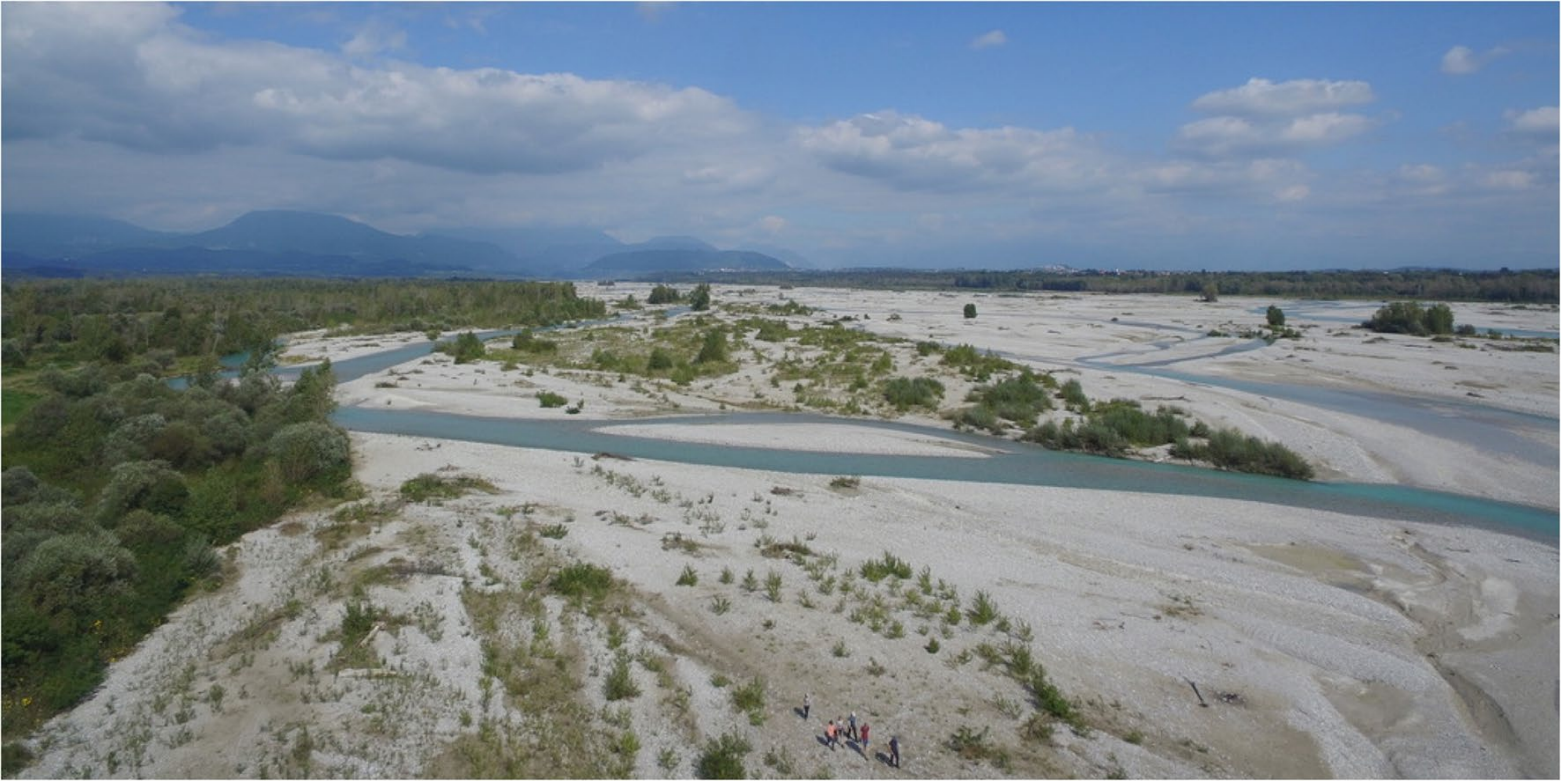
Typical habitat situation in a braided river (Tagliamento) riverscape with a mosaic of different habitat types and large areas of open gravel bars (Photograph Dr. T. C. Wagner).

Natural, unimpaired braided rivers are characterized by exceptionally high habitat dynamics. Existing habitats are occasionally eroded, while elsewhere, new gravel and sand bars are deposited, colonized by pioneer plants, and developed into riparian forests through succession over time^1,2^. The constantly changing mosaic of different habitats (Fig. 1) of such riverscapes^3^ is responsible for their high biodiversity and creates habitats for numerous animal and plant species that are specially adapted to these conditions^4–6^.

Pioneering species typical of braided rivers rely on rapid and efficient colonization strategies for habitat rescue or the conquest of newly created habitats to establish and maintain a stable metapopulation^7–9^. For this purpose, many typical plant species produce a high number of wind-dispersed seeds, germinating and developing into seedlings quickly under favorable conditions^4,10^. The dispersal distance of such species is often comparatively small^11,12^, but under natural conditions, suitable habitat patches mainly occur close to each other. Long-distance dispersal is rare and infrequent^13–15^ as only a tiny fraction of seed lands on the river are transported over longer distances during flood events. When a seed then lands at a site, habitat quality, microsite availability, but also biotic factors such as competition, herbivory and priority effects determine whether the seed can germinate and seedlings eventually establish successfully. While microsite availability and seed pressure play the main role in early colonization, competition and priority effects can largely be neglected^16^ and presence of larger plants is considered in habitat suitability (vegetation coverage and height). Hence, successful colonization requires suitable habitat patches allowing for plant establishment.

For the colonization of new habitat patches, a single established individual is sufficient; once reproductive, its mass of seeds can readily establish in the vicinity of the mother plant and quickly spread over the whole patch, forming a differentiated subpopulation^17,18^. Otherwise (equal conditions assumed), the colonization potential of a species in a particular riverscape is proportional to the amount of seeds and their probability of reaching a new patch^10,19^. This scenario, in turn, depends on the dispersal characteristics of the species, e.g. dispersal distance, the spatio-temporal configuration of suitable habitat patches within the riverscape, the distance of a potential source population to the receiving patch and its shape, and the area^20^. Suppose that a riverscape is to be assessed in terms of the colonization potential for a species in general and independent of the concrete state of the current metapopulation. Therefore, it is necessary to relate the spatial configuration of suitable habitat patches to the dispersal characteristics of the species in question, and to determine the reachability and connectivity of the available suitable habitat patches.

Today, rivers worldwide and in Europe, particularly most braided rivers, are subject to comprehensive regulatory measures and hydropower utilization with severe consequences for biodiversity, ecosystem processes, and services^1,21^. As a result of the regulatory measures, habitats are lost, and the remaining remnants are often significantly fragmented^22^. Consequently, many typical species relying on these habitats are endangered or extinct today^23–25^. Through the European Water Framework Directive^26^ initiative, efforts are being made to restore habitats, revitalize river sections, and reintroduce these species^27^.

However, despite several recent restoration actions, the conservation state of threatened riparian species could not be improved significantly^28,29^. The reasons for this failure are the lack of successful colonization events, habitat dynamics, and habitat fragmentation aspects^30,31^. A wide range of metrics and indices has been developed to quantify the different degrees of connectivity of river systems (for a comprehensive overview, see^32^). However, none of these approaches is suitable for quantifying the colonization potential of a riverscape for usually wind-dispersed plant species typical of braided rivers: the vast majority of these metrics consider only the aquatic component of the riverscape, its network, and its organisms. The focus is mainly on the longitudinal connectivity and passability of fish^33,34^; but see^35^, while the terrestrial components are disregarded. For plants in riverscapes, predominantly genetic isolation by distance and the effects of dams (on community assemblage) have been studied^23,36^. Other connectivity indices for terrestrial ecosystems are almost exclusively focused on animals. If applicable to plants, the particularities of the colonization process and spatio-temporal aspects of terrestrial parts of riverscapes are not considered in such indices. Due to their focus on animals, connectivity metrics for terrestrial ecosystems take into account size, edge, and sometimes in the broadest sense, the shape of a patch^37^. Still, they are primarily focused on large scales and, frequently, the habitat patch is viewed as a homogeneous whole. They do not distinguish whether plants in certain areas of the patch contribute more or less to the connection of neighboring patches.

In addition, the seed exchange probability is mainly considered the resistance of the matrix between two habitat patches, often related to the total landscape area (e.g.,^38^). Even cell-based indices are often unnecessarily complex for our purpose due to their focus on animals (network approach) and not independent of factors, such as total area or occupancy (see^39^). Moreover, these indices mainly refer to habitat connectivity on a landscape scale, and none of these indices is well-suited to assess the colonization potential of an individual habitat patch (for a concise summary, see Supplementary Table A1). A comprehensive overview of terrestrial connectivity indices for conservation is provided by Keeley et al.^40^.

Here, we propose a set of metrics to quantify the potential of a riverscape to be colonized by wind-dispersed plant species. Our metric considers the specifics of dynamic river ecosystems. It allows for a realistic assessment of the colonization potential for a given dispersal characteristic, both at a riverscape and a patch level. The metrics are spatially explicit and can serve as a basis for modeling the situation under a changing spatio-temporal habitat configuration. In addition, they can easily be combined with actual occupancy data, seed rain, and least-cost path approaches. We demonstrate the application of our measures for three differently fragmented riverscapes using the example of two typical braided river species. All metrics were implemented as an R package (“*rconnect*”) which is available through github (https://github.com/TCWagner/rconnect).

## Methods

### Calculation of the metrics

**Figure 2:**
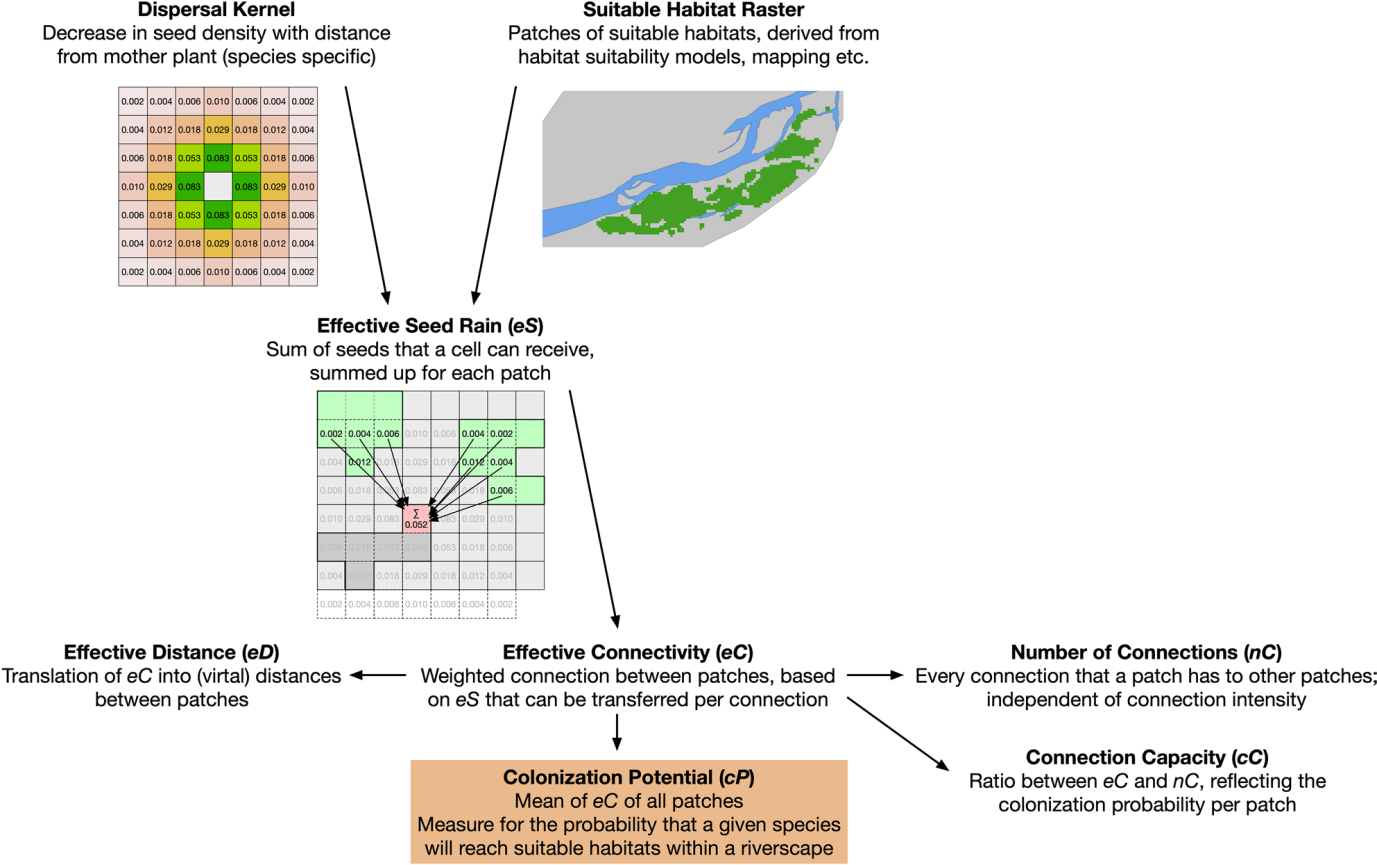
Overview of the relations between the introduced metrics. The species dispersal kernels (as matrix) and suitable habitats (as binary raster) data were used to determine the *effective seed rain (eS)*. This is used to calculate the *effective connectivity* (*eC*), which can be translated into the *effective distance (eD)*, the virtual distance between patches within a riverscape. The *number of connections* (*nC*) and the *connection capacity* (*cC*), as the ratio between the number and strength of connections, characterize the connectivity between patches. The mean of the *eC* of all patches within the riverscape quantifies the species' *colonization potential* (*cP*).

We propose a set of different metrics to describe the potential of a riverscape for its colonization by a wind-dispersed plant species: *effective seed rain* (*eS*), *colonization potential* (*cP*), *number of connections* (*nC*), *effective connectivity* (*eC*), and *effective distance* (*eD*). As input data, a raster with suitable habitats and a matrix with the dispersal kernel (or dispersal distance) of the species are required. All metrics are available at the patch level, *cP* and *eD* are available on a riverscape scale, and *eS* is available at the raster cell level (Fig. 2). Calculation of our metrics was done in R^41^, using the *raster* package^42^ and implemented as the R package *rconnect*.

#### Basic considerations

Our metrics only distinguish between suitable habitats and those that are fundamentally unsuitable. The actual occupancy or weighted habitat suitability is not considered. All suitable patches can be both potential donor and recipient habitats from or to which colonization events occur. A patch consists of a contiguous group of cells separated from other patches by unsuitable cells. Each patch cell is considered individually for our analysis. Furthermore, we assume that one (successfully) colonized cell is sufficient to establish a subpopulation on the respective suitable patch. The probability of such a colonization event is directly proportional to the number of seeds reaching this patch and is the sum of all seeds from the cells of suitable neighboring habitats that reach the cell of the receiving habitat. The relative amount of seeds each cell in a suitable habitat patch may receive in the given habitat configuration is compared to an ideal cell (a cell that receives all the seeds produced by a plant or population). Any cell receiving as many seeds as this ideal cell is set to value 1 (probability; maximum contribution to colonization). Cells that do not receive any seed or are unsuitable for the establishment of the species will be represented by 0. In a real environment, each cell will only receive a fraction of the number of seeds, depending on the number of actual donor cells, their distance to the receptor cell, and the species dispersal kernel.

#### Dispersal kernel

The species dispersal kernel is the two-dimensional probability density function representing the seeds falling in the distance from a source and assuming an equal probability of traveling in all directions. The short distance dispersal range of a species (SDD) is usually the radius around a mother plant within which 95% (or 99%) of the seeds land^43^. The calculation base is the dispersal characteristics of the respective species, usually a negative exponential decrease with distance. We implement the dispersal kernel as a two-dimensional square matrix with an odd number of rows and columns, the center element representing the focal cell for which the number of incoming seeds is to be determined. The fraction of seeds received at a certain distance is thereby:

$${\text{sc}} = (1 - decay)^{distance}$$

For practical reasons, the number of rows/columns of the dispersal kernel matrix needs to consider the spatial resolution of the habitat patch raster. The number of rows/columns should be as small as possible but at least cover the two-fold dispersal distance of the species. Given a habitat raster with five-meter cell size and a species dispersal distance of 15 m, the kernel matrix should at least have a dimension of (2 × 15)/5, i.e., six rows and columns. As the number needs to be odd, the final kernel matrix would consist of seven rows and seven columns.

**Figure 3:**
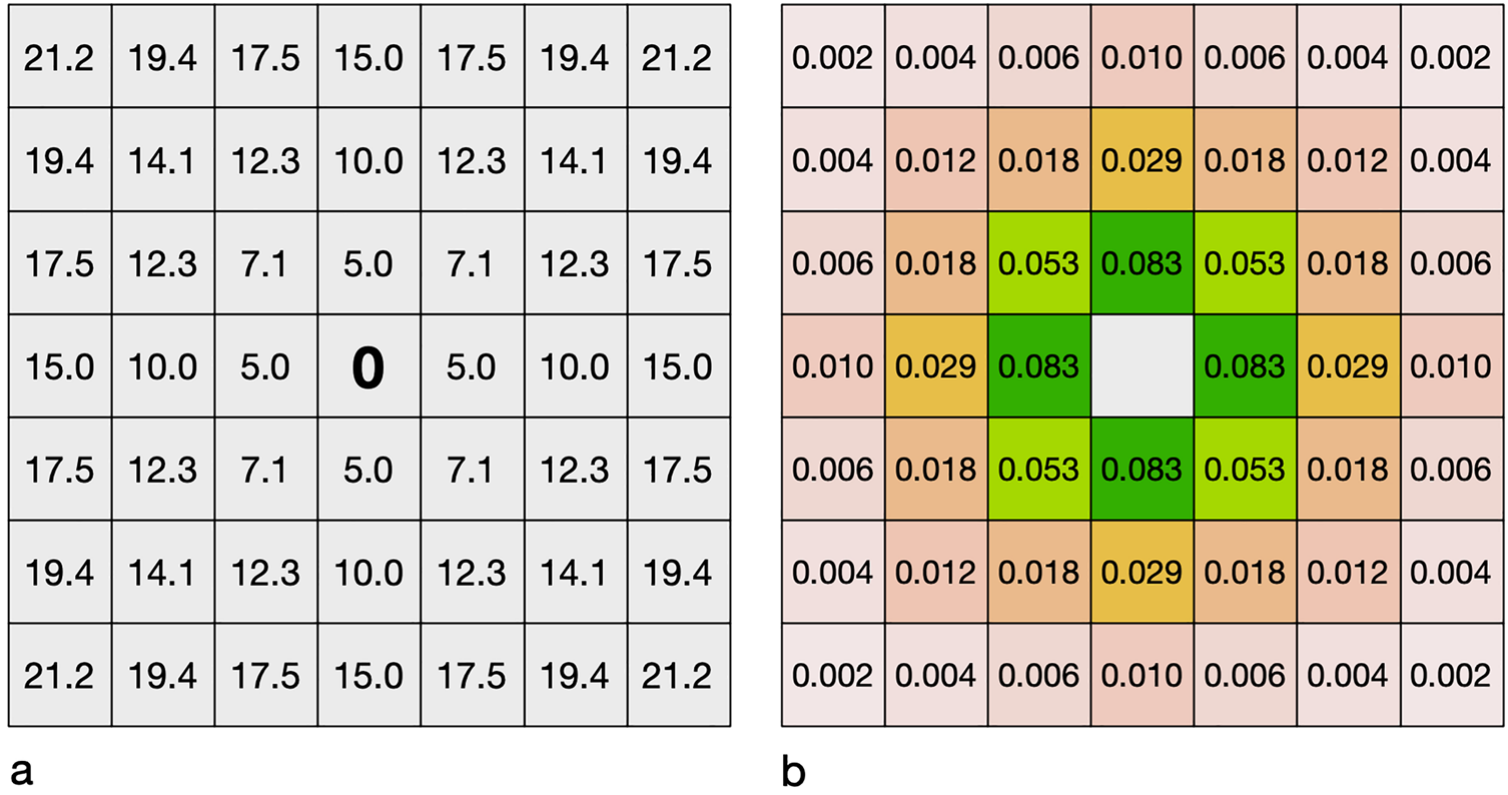
Exemplary dispersal kernel of *Chondrilla chondrilloides* with a radius of three cells (7 × 7 elements), matching a raster width of 5 m. (**a**) distances between the center of the focal cell (0) and the respective cell; (**b**) the relative proportion of seeds each cell theoretically contributes to the focal cell under ideal conditions (sum = 1).

According to the distance of the respective cell to the center, it is first calculated for each cell what proportion of seeds from it will land on the central cell under ideal conditions. The value of the center element is set to 0 (Fig. 3), and the matrix is divided by its sum to normalize the matrix. This ensures that the total sum of all cells is 1, i.e., that 100% of all potentially possible seeds reach the center cell:

$${\text{s}} = (1 - decay)^{distance} /\sum (kernel\;matrix)$$

**Figure 4:**
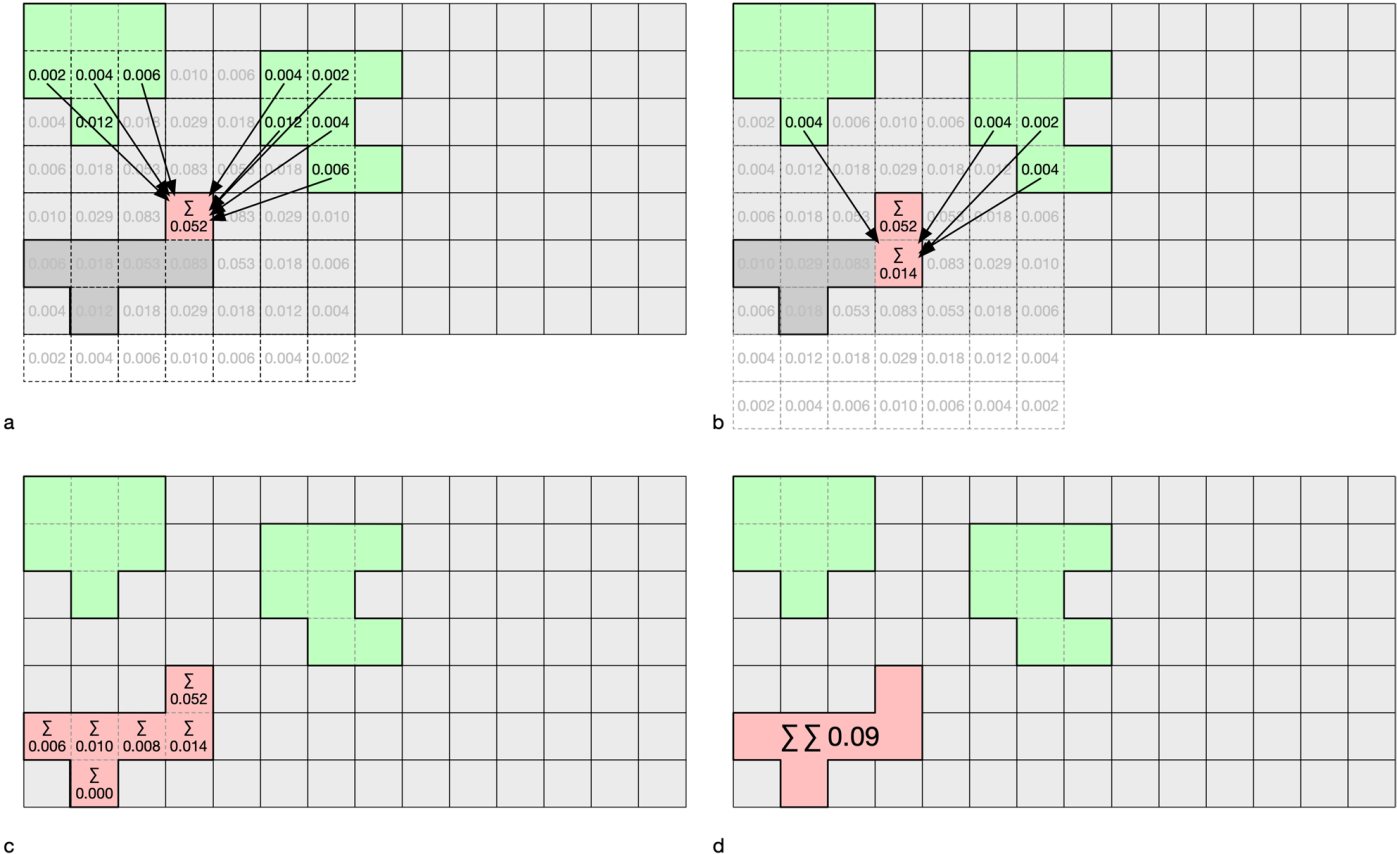
Applying the moving kernel window to determine the relative colonization potential, expressed as a fraction of seeds each cell receives from its neighbors within the species dispersal distance. (**a**,**b**) The respective focal cells (red) receive 0.052 and 0.014 times the amount of seed that an ideal cell would receive. (**c**,**d**) Sums of individual cells and the total sum of seeds received by the patch.

This kernel matrix (Fig. 3) is then applied as a focal window to the raster containing suitable habitats (Fig. 4).

#### Effective seed rain (eS)

The core of our considerations is the relative amount of seeds that can be exchanged between neighboring patches according to the dispersal characteristics of a species. Under otherwise equal conditions, the probability that a patch will be colonized is directly proportional to the number of seeds that reach it (twice as many seeds means twice the probability that the patch will be colonized). Since the reachability of seeds is bidirectional, this means that this patch also has twice the potential to colonize other patches. This potential is expressed by the *effective seed rain (eS)* measure. This metric specifies for each grid cell (receiving cell) of a suitable habitat patch the theoretical proportion of seeds that this cell will obtain from the cells of the other suitable habitat patches (donor cells). Values for *eS* reach from 0 to 1, where 0 means that the receiving cell does not receive any seeds, and 1 means that the amount of seeds received is identical to the amount the donor cell itself would receive. Suitable cells on the same patch as the receiving cell are not considered donor cells. The number of seeds reaching the receiving cell is determined by the dispersal kernel and the distance between the donor and the receiving cells (Fig. 4).

**Figure 5:**
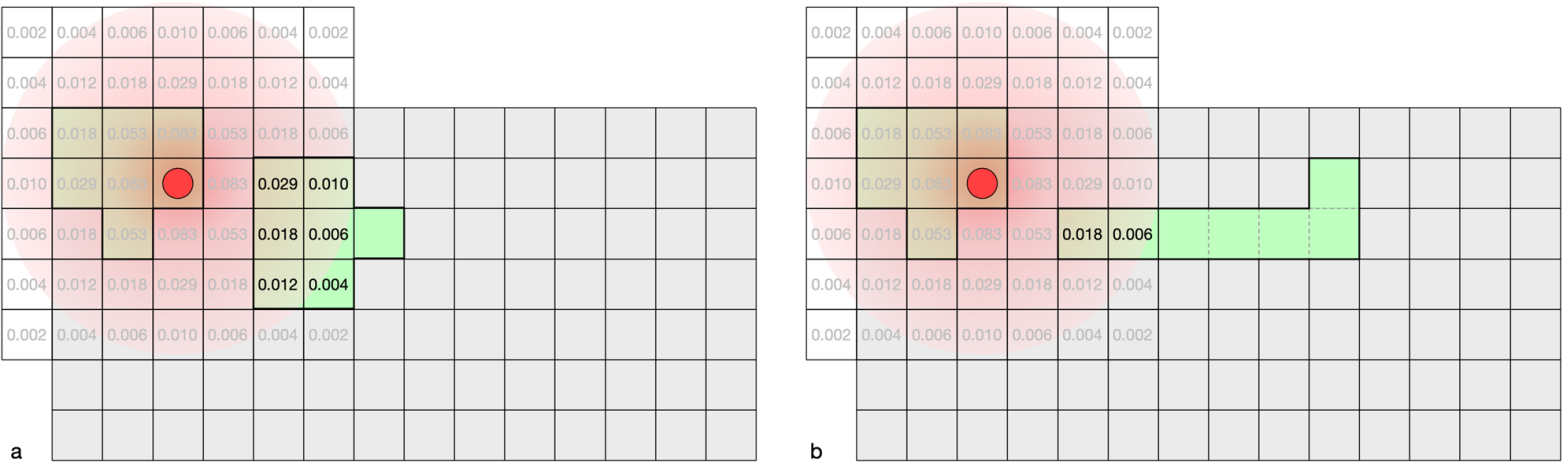
Effect of patch shape on the relative amount of seeds received from patches of equal area.

For the given dispersal kernel of a species, the distance and influence of each individual cell of a patch are taken into account, as well as the size and shape of the patch. *Effective seed rain* can differ considerably between patches of the same size but different shapes (Fig. 5).

In the ideal case, the receiving cell is within the dispersal distance of the species and is surrounded exclusively by suitable cells. Then, the proportion of seeds would add up to 1, indicating that this cell has the highest possible colonization potential. Therefore, *eS* provides spatially explicit the relative probability with which the receiving cell can be colonized, including the spatial configuration of suitable patches within the riverscape. The higher the *eS* value of a cell, the higher the potential for the individual cell to become colonized. As this measure works in both directions, it also means that a population in such a cell has a high potential to colonize neighboring patches.

#### Effective connectivity (eC) and colonization potential (cP)

The colonization probability of an entire patch is expressed by the measure of *effective connectivity* (*eC*). The *eS* values of all cells of a discrete suitable habitat patch are summed up to calculate *eC* (Fig. 4c,d). Depending on the size of the receiving habitat, the number of donor cells, and the extent to which they contribute seeds, values above 1 are possible. The resulting measure is linear, i.e., twice the value means double colonization potential. A patch with a value of 2.5 is 2.5 times more likely to become colonized or colonize other patches than a patch with a value of 1. The *eC* can also be specified for explicit patch-patch relationships to characterize the relative strength of the connection between these patches. The mean of the *eC* values of all suitable habitat patches of a given riverscape describes the *colonization potential* (*cP*) of the respective riverscape. Under otherwise equal conditions, for any given spatial patch configuration with a *cP* of 0.5 (for a certain species), five times more seeds are likely to reach a suitable patch than in a configuration with a *cP* of 0.1.

#### Effective distance (eD, eDm)

Based on the *eC*, it is possible to calculate the *effective distance* (*eD*). This results from the amount of seeds that a patch can receive from all other patches of the riverscape converted to the distance that would correspond to this amount of seed for the given dispersal kernel. This virtual distance is, therefore, the log of the *eC* value to the base of 1- decay of the negative exponential dispersal kernel:

$${\text{eD}} = {\text{log}}_{{({1} - {\text{decay}})}} (eC)$$

*Effective Distances* < 0 were set to zero to avoid a negative *eD* for *eC* values higher than 1. The *eD* values of all connected patches within a riverscape can be averaged to obtain a mean *eD* (*eDm*) for the entire riverscape. The *effective distance* (*eD, eDm*) can be directly related to the dispersal distance of the species: *eD* values above the dispersal distance of the species make a colonization event for the given patch or riverscape unlikely, and colonization probability increases exponentially with decreasing *eD*.

#### Number of connections and connection capacity (nC, cC)

Due to the high dynamics of the habitat situation and the perpetual rearrangement of habitats in braided rivers, risk spreading is also essential for successfully establishing and maintaining a stable metapopulation. Patches that receive seeds from several surrounding patches or that can pass seeds on to many neighboring patches increase the probability of establishing a metapopulation. Patches with only a few connections might face a higher risk of either donor or receiving patches being lost. We apply the *number of connections* (*nC*) and *connection capacity* (*cC*) to characterize the connectivity of each patch in terms of seed reachability. Both are available at the patch and riverscape levels (*nCm* and *cCm*). The *number of connections (nC)* specified for each patch and how many other patches it could exchange seeds, regardless of the strength of the respective exchanges. The mean of all *nC* values within the riverscape (*nCm*) provides its average connectivity.

The capacity of the respective connections determines the relative amount of seeds that can be transferred via this connection, and hence affects the colonization probability. For this purpose, the *eC* of a patch is divided by *nC* to obtain the average *connection capacity* (*cC*). For example, if a patch is connected to two other patches (*nC* = 2) and *eC* to the first patch is 0.5, to the second, 0.1, *cC* is the (0.5 + 0.1)/2, that is 0.3.

### Example riverscapes and example species

**Figure 6:**
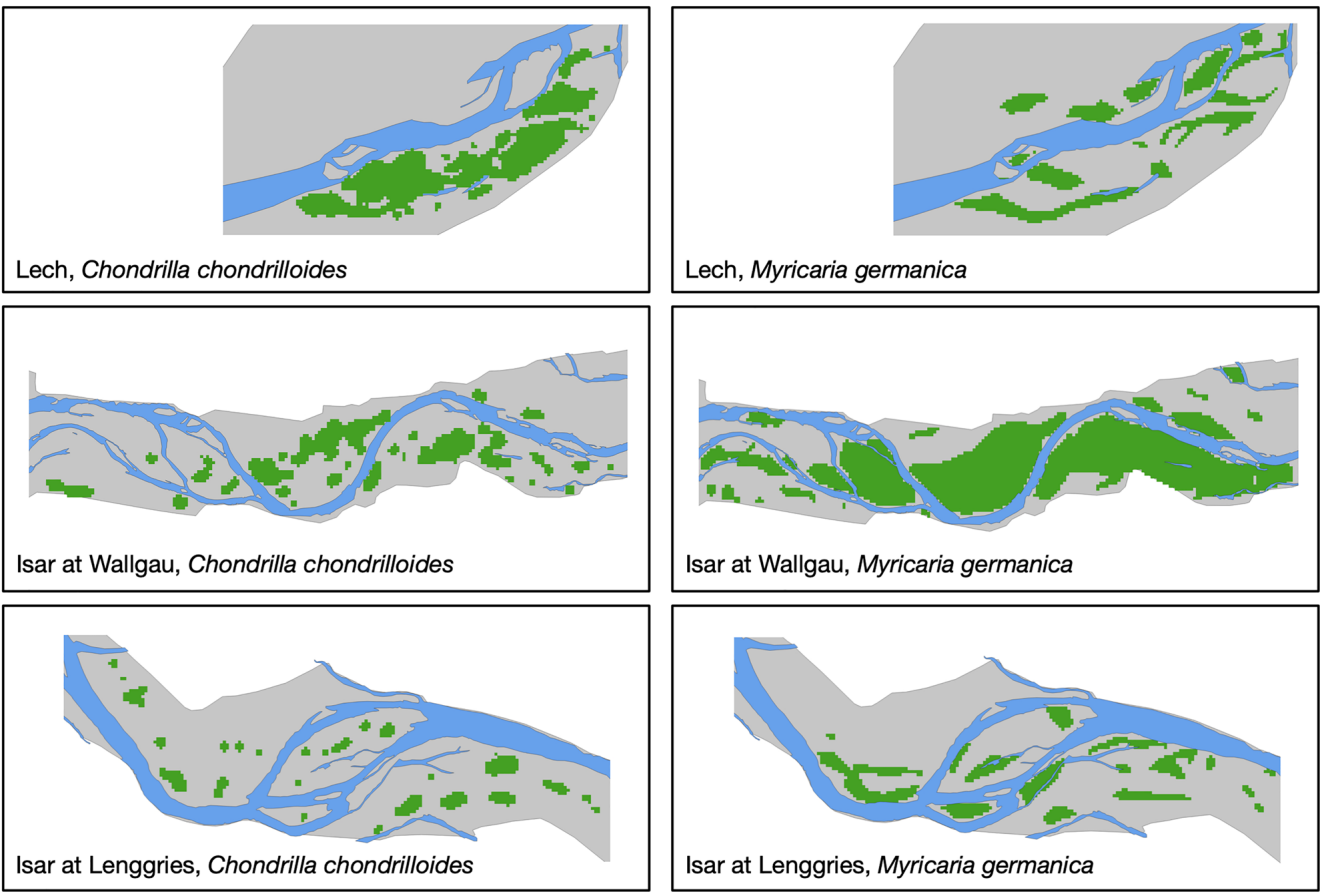
Example riverscapes at the Tyrolean Lech and Isar with different configurations of suitable habitat patches for *Chondrilla chondrilloides* (left) and *Myricaria germanica* (right). Suitable habitat patches were determined using a surface range envelope model for the respective species. *M. germanica* still occurred along all study sites. Natural populations of *C. chondrilloides* are found only at the Tyrolean Lech.

As example riverscapes, we chose a near-to-natural section of the Tyrolean Lech River (32T 623000E 5254680N), a largely unaffected section of the Isar River between Wallgau and Vorderriss (32T 680850E 5269100E)), and a regulated and straightened section of the Isar River at Lenggries (32T 694150E 5276250N)^44,45^. Habitat suitability was derived from respective habitat suitability models for both species to obtain a realistic habitat configuration for each species (Woellner & Wagner, unpublished). All riverscapes differed in their spatial configuration and the sizes of potential habitat patches for the example species (Fig. 6).

We chose *Chondrilla chondrilloides* (Ard) H. Kast (Asteraceae) and *Myricaria germanica* (L.) Desv.(Tamaricaceae) as the study species. Both species are typical representatives of alpine river specialists and character species of natural braided rivers, but differ somewhat in their habitat requirements^12,24^. Before human intervention in the last century, both species occurred along most alpine rivers. Both study species were wind-dispersed with a negative exponential dispersal kernel. *Chondrilla chondrilloides* has a dispersal distance of 14 m^12^, corresponding to a decay of 0.19 for a negative exponential dispersal kernel. *Myricaria germanica* has a dispersal distance of 30 m^46^ and a decay of 0.10. The germination rate of both species is > 95%^12,24^. Both species have been present within these riverscapes until the last century. Today, *C. chondrilloides* occurs only at the Tyrolean Lech^12^. *Myricaria germanica* still occurs in all example riverscapes^25^, although only sporadically in the Lenggries section.

All riverscapes were represented as raster data with a 5-m cell size in a metric Cartesian coordinate system (e.g., UTM). Cells with suitable habitats were coded with 1, unsuitable cells with 0, and no weighting of habitat suitability was considered. Areas outside the active river channel were coded with NA.

## Results

**Figure 7:**
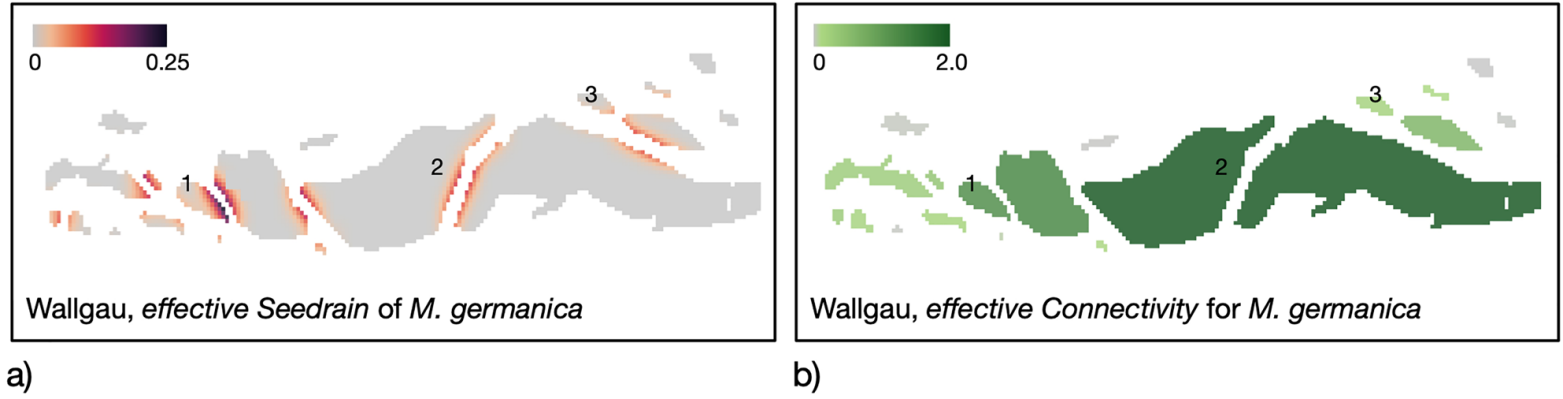
(**a**) Example of the *effective Seed rain* (*eS*) for *Myricaria germanica* within the Wallgau riverscape. 1: patch with a medium-large contact area to several neighboring patches, one relatively close; 2: patch with a large contact area, but neighboring patches are relatively far from each other; 3: patch with only a contact area. (**b**) resulting in *effective connectivity* (*eC*) at the patch level.

Applied to the example riverscapes, our metrics adequately reflect the availability and spatial configuration of suitable habitat patches for the different example species. The *effective seed rain* (*eS*) spatially explicitly represents the situation, considering the topology and spacing of patches (Fig. 7) and takes into account the shape and size of the respective interface and the distance between patches. Areas of patches from which seeds cannot reach a neighboring patch are ignored. They do not contribute to the colonization potential although, of course, propagation of the species may also occur within the patch.

At the patch level, it is possible to identify those patches within a riverscape with the most connections and for which these connections have a high *connection capacity (cC)*. The relative standard deviation of the respective values shows how uniformly the colonization potential is distributed within the riverscape (Tables 1, 2). The *effective distance (eD)* can be directly related to the dispersal distance of a species and allows an assessment of the relative proportion of seeds that will reach a patch.

**Table 1:** Values of *effective connectivity* (*eC*), *effective distance* (*eD*), *number of connections* (*nC*), and *connection capacity* (*cC*) of our example riverscapes for *Chondrilla chondrilloides* at the patch level*.* The threshold for *eC, nC*, and *cC* is 0.01.

Patch	*Chondrilla chondrilloides* *SDD* = 14 m											
	Lech				Wallgau				Lenggries			
	*eC*	*eD*	*nC*	*cC*	*eC*	*eD*	*nC*	*cC*	*eC*	*eD*	*nC*	*cC*
1	0.00	25.12	0	0.00	0.00	42.18	0	0.00	0.00	24.49	0	0
2	0.38	4.56	1	0.38	0.00	47.03	0	0.00	0.01	18.96	1	0.01
3	1.90	0.00	3	0.63	0.47	3.49	4	0.12	0.01	20.73	1	0.01
4	0.11	10.53	2	0.05	0.41	4.27	2	0.20	0.00	39.82	0	0.00
5	3.05	0.00	6	0.71	0.41	4.28	3	0.14	0.00	39.82	0	0.00
6	0.16	8.74	1	0.16	0.03	14.84	1	0.03	0.00	∞	0	0.00
7	0.37	4.74	1	0.37	0.11	9.93	1	0.11	0.09	11.45	1	0.09
8	0.98	0.09	1	0.98	0.00	∞	0	0.00	0.09	11–19	1	0.09
9	0.04	15.59	1	0.04	0.52	3.07	2	0.26	0.00	25.17	0	0.00
10					0.12	10.04	2	0.06	0.00	∞	0	0.00
11					0.00	21.94	0	0.00	0.00	∞	0	0.00
12					0.00	23.40	0	0.00	0.00	23.03	0	0.00
13					1.02	0.00	3	0.34	0.16	8.46	1	0.16
14					0.09	11.25	2	0.05	0.16	8.53	1	0.16
15					0.00	19.16	0	0.00	0.03	16.26	1	0.03
16					0.00	24.57	0	0.00	0.00	31.59	0	0.00
17					0.00	35.20	0	0.00	0.03	17.13	0	0.03
18					0.07	12.70	2	0.03	0.00	27.49	0	0.00
19					0.00	20.06	0	0.00	0.00	32.59	0	0.00
20					0.05	14.23	1	0.05	0.00	31.13	0	0.00
21					0.00	∞	0	0.00	0.00	23.85	0	0.00
22					0.00	21.61	0	0.00	0.00	23.85	0	0.00
23					0.69	1.77	2	0.34	0.00	28.10	0	0.00
24					0.00	24.49	0	0.00	0.08	12.00	1	0.04
25					0.00	30.51	0	0.00	0.08	12.16	1	0.08
26					0.08	12.24	1	0.08				
Mean	0.78 ± 1.04	7.71 ± 8.44	1.78 ± 1.79	0.39 ± 0.32	0.16 ± 0.27	17.18 ± 12.63	1.00 ± 1.20	0.14 ± 0.11	0.03 ± 0.05	22.17 ± 9.43	0.40 ± 0.50	0.07 ± 0.06
%sd	1.35	1.09	1.01	0.82	1.69	0.74	1.20	0.81	1.67	0.43	1.25	0.75
%con			88.9				50.0				36.0	

Mean ± SD, relative SD (%SD), and percent connected patches (%con) are given. The mean *eC* corresponds to the *effective connectivity* on the riverscape scale (*eCm*). Values of the (virtual) *effective distance* above the dispersal distance (14 m) are highlighted (red), as they result in zero connections. These patches do not contribute to the connectivity of the riverscape.

**Table 2:** Values of *effective connectivity* (*eC*), *effective distance* (*eD*), *number of connections* (*nC*), and *connection capacity* (*cC*) of our example riverscapes for *Myricaria germanica* at the patch level.

Patch	*Myricaria germanica* *SDD* = 30 m											
	Lech				Wallgau				Lenggries			
	*eC*	*eD*	*nC*	*cC*	*eC*	*eD*	*nC*	*cC*	*eC*	*eD*	*nC*	*cC*
1	1.61	0.00	2	0.80	0.00	79.79	0	0.00	0.12	21.38	1	0.12
2	3.52	0.00	3	1.17	0.19	16.63	1	0.19	0.12	21.38	1	0.12
3	2.75	0.00	3	0.92	0.38	9.78	2	0.19	0.00	∞	0	0.00
4	0.21	15.63	1	0.21	0.01	44.18	1	0.01	0.35	10.63	1	0.35
5	4.59	0.00	3	1.53	0.00	49.96	0	0.00	0.35	10.42	1	0.35
6	0.00	77.72	0	0.00	5.30	0.00	3	1.77	0.11	21.79	1	0.11
7	0.21	15.61	1	0.21	5.49	0.00	3	1.83	0.11	22.45	1	0.11
8	3.64	0.00	3	1.21	2.28	0.00	3	0.76	0.00	∞	0	0.00
9	0.07	27.10	2	0.03	0.00	53.58	0	0.00	0.67	4.05	2	0.33
10	0.11	21.97	2	0.06	5.73	0.00	4	1.43	0.18	17.34	2	0.09
11	0.68	3.80	3	0.23	1.30	0.00	5	0.26	0.13	20.01	2	0.07
12	0.08	25.15	3	0.03	1.04	0.00	3	0.35	0.90	1.02	3	0.30
13	1.29	0.00	3	0.43	4.30	0.00	4	1–08	0.19	16.67	1	0.19
14	1.04	0.00	2	0.52	0.54	6.18	2	0.27	0.16	18.35	2	0.08
15	1.65	0.00	3	0.55	0.53	6.28	3	0.18	0.15	19.26	1	0.15
16					0.67	3.88	3	0.22	0.01	42.84	1	0.01
17					0.01	40.87	1	0.01				
18					0.08	25.49	2	0.04				
19					0.26	13.67	2	0.13				
Mean	1.43 ± 1.52	12.47 ± 20.81	2.27 ± 0.96	0.56 ± 0.49	1.48 ± 2.07	18.43 ± 23.80	2.21 ± 1.44	0.54 ± 0.63	0.22 ± 0.25	17.68 ± 9.90	1.25 ± 0.77	0.17 ± 0.11
%sd												
% conn			93.3				84.2				87.5	

The threshold for *eC, nC,* and *cC* is 0.01. Mean ± SD, relative SD (%SD), and percent connected patches (%con) are given. The mean *eC* corresponds to the *effective connectivity* on the riverscape scale (*eCm*). Values of the (virtual) *effective distance* above the dispersal distance (14 m) are highlighted (red), as they result in zero connections.

Calculated as patch-patch relations, *eC* and *eD* both measures allow the assessment of individual relationships between patches (for an example see Supplementary Fig. A1; Tables A2, A3).

At the riverscape level, our metrics provide an intuitive way of assessing the colonization potential of the entire riverscape. It allows for a direct comparison between different species and riverscapes with different spatial configurations of suitable habitats (Table 3). The relative standard deviations allow for estimating the relative variation between patches.

**Table 3:** Metrics at the riverscape level: *colonization potential* (*cP*), mean *effective distance* (*eDm*), mean *number of Connections* (*nCm*), and average *capacity of connection* (*cCm*) of the differently fragmented example riverscapes, and the relative standard deviation in brackets.

Riverscape		*Chondrilla chondrilloides* SDD = 14 m				*Myricaria germanica* SDD = 30 m			
		*cP*	*eDm*	*nCm*	*cCm*	*cP*	*eDm*	*nCm*	*cCm*
1	Lech	0.78 (1.33)	7.71 (1.09)	1.79 (1.00)	0.39 (1.10)	1.43 (1.06)	12.47 (1.67)	2.27 (0.42)	0.56 (0.88)
2	Wallgau	0.16 (1.69)	17.18	1.00	0.14	1.48 (1.40)	18.43 (1.23)	2.21 (0.65)	0.54 (1.17)
3	Lenggries	0.03 (1.64)	22.17	0.40	0.07	0.22 (1.14)	17.68 (0.56)	1.25 (0.62)	0.17 (0.65)

The studied species, *Chondrilla chondrilloides* and *Myricaria germanica* have dispersal distances of 14 m (decay 0.19) and 30 m (decay 0.10), respectively.

Based on a threshold of 0.01 (corresponding to 1% of the seeds), for *C. chondrilloides* (Table 1), the overall patch connectivity and the average number of connections (*nCm*) were highest in the Lech riverscape (89% connected patches, *cC* = 0.39, *nCm* = 1.78), intermediate in Wallgau (50%, *cC* = 0.14, *nCm* = 1), and lowest in the Lenggries riverscape (36%, *cC* = 0.07, *nCm* = 0.4). For *M. germanica* (Table 2), patch connectivity was consistently high for all riverscapes. It was highest in the Lech riverscape with 93% connected patches (*cC* = 0.56), followed by Wallgau, and Lenggries riverscapes with 84% (*cC* = 0.54) and 87% (*cC* = 0.17), respectively. While the average *capacity of Connections* (*cCm*) for Lech and Wallgau were about the same, the *cC* of Lenggries was the lowest.

For *C. chondrilloides,* the *colonization potential (cP)* was highest for the Lech riverscape with 0.78, *cP* for the Wallgau riverscape was 4.9 times lower, and even 26 times lower for the Lenggries riverscape. The average *effective distance* (*eDm*) of ~ 8 m for the Lech riverscape was well below the species dispersal distance of 14 m. However, for the Wallgau riverscape, *eDm* at 17 m was slightly above, and for the Lenggries riverscape at 22 m, it was above the dispersal distance (Table 3). The *colonization potential (cP)* for *M. germanica* is high for the Lech and Wallgau riverscape, with 1.43 and 1.48. Despite the high number of connected patches, *cP* of the Lenggries riverscape was considerably lower, with only 0.22, roughly 6.5 times lower than for the Wallgau and Lech riverscapes. The average *effective distance* (*eDm*) was well below the species dispersal distance for all riverscapes, but the lowest at the Lech (Table 3). The high relative standard deviation suggests that the colonization potential of the individual patches is unevenly distributed throughout the riverscapes.

A brief comparison of the results of our metric with some commonly used distance and connectivity measures, such as edge-to-edge nearest neighbor distance or landscape coincidence probability (LCP), is provided in the appendix (see Supplementary Tables A4 and A5).

## Discussion

Our metrics fulfill several essential requirements for assessing and modeling the colonization potential of riverscapes. Most measures are available at both the patch and landscape levels, and *eS* is even available at the cell level. The results are spatially explicit and can be combined with other raster-based methods, such as multiplication with weights (e.g., for population densities, habitat quality or barriers). When iterated over several timesteps, the population dynamics for a specific riverscape can be modeled (cf.^47^). The metrics consider the specific interface between connected patches, are easily translatable regarding colonization potential, and provide a realistic ranking. The metric values allow a direct comparison of riverscapes with different spatio-temporal habitat patch configurations, even though these values depend on the particular dispersal characteristics of a species. All values are linear representations, i.e., a value twice as high means twice as high colonization potential.

The *effective connectivity* (*eC;* patch scale) or the *colonization potential (cP;* riverscape scale) are measures of the probability with which a patch or riverscape can be colonized, which is an essential factor for the establishment and maintenance of stable metapopulations. An *eC* value of 1 means that the respective patch potentially receives as many seeds as a donor cell produces under ideal conditions, and that the relative probability of a seed reaching the patch is 100%. The *eC* of a patch represents a measure of the colonization potential of this patch, and the average *eC* of all patches within a riverscape reflects the colonization potential *cP* of the entire riverscape for the respective species.

The *effective distance* (*eD* can be directly related to the dispersal distance of a species, thereby allowing a quick assessment of its colonization probability in the given riverscape. If the average *effective distance* of the riverscape (*eDm*) is above the species dispersal distance, this means that, in this riverscape, on average, less than 5% of the seeds of potential donor cells can reach a patch. Hence, successful colonization events are rare or unlikely. As *eDm* for a riverscape is calculated only for connected patches (not connected patches would result in an infinite *eD*), *eDm* needs to be interpreted together with the percentage of connected patches.

A higher number of connections *nC*, a higher connection capacity *cC,* and a balanced distribution of these connections provide redundancy and mean risk spreading and reduce the probability of species extinction^48^, particularly in the highly dynamic environments of braided rivers. A higher number of connections reduces the risk that available donor cells (respectively receiving cells) or even their whole patches will become eradicated during habitat turnover. An increased connection capacity means the transfer of a higher amount of seed and hence a higher colonization probability provided by the available connections.

### How well do the metrics reflect the situation of the example riverscapes?

For *C. chondrilloides*, the values of our metrics suggest a high colonization potential for the Lech riverscape. In contrast, colonization of the Wallgau riverscape is less likely, as the smaller and partly more isolated patches have an *eD* slightly above the species dispersal distance, resulting in a lower *cP* and *cC.* In the Lenggries riverscape, *eD* is clearly above the dispersal distance, patches are only connected to a small extent, and the capacity of the connections is very low, making colonization unlikely. As the patch size decreases and the distance between patches increases, the *colonization potential (cP)* clearly decreases. The results reflect today's distribution of the species, where a natural and large metapopulation of *C. chondrilloides* is found only in the Lech riverscape. In the Wallgau riverscape, a small population was reintroduced in 2018 and initially increased on the local patch but did not colonize neighboring patches^12^ and in the Lenggries riverscape the species is missing.

In comparison, the colonization potential of *M. germanica* is substantially higher in all riverscapes. *M. germanica* is less susceptible to fragmentation due to its greater dispersal distance and, therefore, shows higher colonization potential even in more scattered habitat situations with smaller patch sizes. This can be seen in the Lech riverscape, where the habitat situation appears to be much more fragmented than in the Wallgau riverscape. Nevertheless, the *cP*, *nC,* and *cC* of these riverscapes are essentially identical. The importance of the interface and the resulting number and capacity of connections between the patches becomes apparent when comparing the Lech and Lenggries riverscapes. Although the habitat situation appears to be only slightly different, the colonization potential of the two landscapes differs considerably. In the Lenggries, the colonization potential of *M. germanica* is more than six times lower, a consequence of the lower number of connections combined with a lower capacity. Hence, even slight differences in the patch configuration and the shape of the interface between patches can result in considerable connectivity differences. Notable rejuvenating subpopulations of *M. germanica* are found only along the Lech River and in the Wallgau riverscape today^12,25,49^.

### Application in the assessment of the ecological status of riverscapes

Currently, the assessment of the ecological status of rivers is mainly based on structural properties and longitudinal connectivity^26,50^. If terrestrial components are considered, the presence of specially adapted plant species, such as *M. germanica*, is often used as an indicator of the condition of a river in terms of ecological processes^24,51,52^. However, due to the frequent habitat turnover in braided rivers, colonization time-lags lead to a high proportion of suitable but unoccupied habitats. Hence, the occupancy of the species does not necessarily reflect whether the riverscape meets the requirements for a stable metapopulation. On the other hand, potentially suitable habitat patches (occupied or not) can be easily identified through appropriate habitat suitability models or habitat surveys. The resulting spatial configuration of suitable habitats can then be assessed using our metrics. Looking at *cP* over a more extended time enables semi-quantitative assessments of changes in the colonization potential of a riverscape over time, allowing conclusions about the success of conservation or restoration measures. A decrease in *cP* indicates an increasing degradation of the riverscape regarding habitat reachability. Similarly, an increase in *eD* indicates a decreasing potential. If *eD* exceeds the species’ dispersal distance, the establishment of a stable metapopulation becomes unlikely. Together with already common criteria, such as structural features, fish passability, habitat availability and quality, and general habitat connectivity, the *cP* of a riverscape for a selected target species can contribute to a better, more comprehensive assessment of the ecological condition of braided rivers.

### Application in restoration and reintroduction measures of riverscapes

The metrics allow for assessing whether the spatial configuration of newly created habitats by restoration measures is suitable for establishing the intended plant target species. Over time, the temporal development of *eDm* and *cP* provides information about a possible improvement or deterioration of the situation.

Many reintroductions proved unsuccessful over a more extended time^28,29^. This also applies to the reintroduction of river specialist plant species^24,25^, where reintroduction often occurs at remote and small-scale restoration sites (see^53^). In principle, reintroduction measures aim to create a self-sustaining metapopulation^54^. At the riverscape level, *eDm* and *cP* provide a reasonable basis for assessing the suitability of a riverscape for reintroduction measures. The smaller the *eDm*, the more likely a successful colonization of connected patches is. If *eDm* of the riverscape exceeds the dispersal distance of the reintroduced species, colonization is unlikely.

The *colonization potential* (*cP*) can be used to assess how likely it is that the species to reintroduce will be able to colonize the respective riverscape. Applying *cP* to different parts of a larger riverscape can help select the most suitable region for reintroduction. Once the general suitability of the riverscape has been established, *cP* and *eD* at the patch level help to identify those patches with the highest potential for initial reintroduction. Ideally, this is made on patches with high *effective connectivity* (*eC*), many connections to neighboring patches (high *nC*) with a *high connection capacity* (*cC*). This ensures the highest propagation probability in the given riverscape and simultaneously spreads the extinction risk of the newly colonized patches.

### Modeling specific situations and dispersal

Our metric, similar to other connectivity indices, represents a general, comparable measure with which the potential of a riverscape to become colonized can be quantified., Beyond, our approach also allows for assessing the actual, more specific situation or for modeling the concrete dispersal of a plant species within a riverscape. This requires the inclusion of further factors, such as the actual occupancy of the donor cells, the demographics and actual seed production of the population, biases in the dispersal kernel due to prevailing wind directions, or graduated habitat quality^55^. For this the effective seed rain (*eS*) can be multiplied by, for example, a grid with relative plant densities, proportion of habitat suitability, or “costs” for a certain patch (see e.g.^56^) and replace the dispersal kernel with an own matrix, reflecting the specific situation (cf.^47^). Similarly, *effective connectivity* (*eC*) can be combined with data on population size or graduated habitat suitability of the respective patches. Our metric also provides the necessary basic data for modeling secondary dispersal via water, since e.g. effective seed rain allows to quantify where a number of seeds will reach ‘water’ cells for potential transport downstream (see^57^).

When using the spatially explicit raster data of our metrics as basis for further dynamic modeling (e.g., by cellular automata models) or state transition models, cost paths and barriers can be included as separate raster data in order to moderate the probability with which individual cells can be colonized.

Hence, the above-mentioned methods allow for an explicit and realistic prediction of the actual colonization processes in the riverscape or the simulation of scenarios with different habitat configurations. These modeling efforts can also be extended to other wind-dispersed species, for example, invasive plants, such as *Buddleja davidii*, *Ailanthus altissima,* and others.

### Limitations

The *colonization potential (cP)* is a comparable measure of the relative probability with which a colonization event happens within a riverscape. However, *cP* does not allow us to predict to what extent and when a riverscape will be populated, nor does it provide the actual probability of colonization. For the latter purpose, *cP* needs to be combined with additional data, such as habitat quality, actual occupancy, fecundity, etc.^55^, but this can be done easily. Currently, our metrics do not consider potential barriers. Due to the raster-based approach, this can easily be implemented by including weights or combining the focal window with cost paths, considering barriers^47^. However, barriers play little role in detailed, small-scale dispersal on the open gravel areas of braided river ecosystems.

Further, our metrics do not account for long-distance and secondary dispersal. Such long-distance dispersal is considerably rarer and, though essential for the colonization of new faraway sites^58,59^, the large-scale spread of the species takes place mainly via primary dispersal^13,58^. With increasing fragmentation, long-distance dispersal might become more critical^59^. However, long-distance dispersal comprises a complex set of completely different processes that can vary considerably between different species. Such processes have to be implemented by other raster based models (e.g. cellular automata based approaches). However, if such models are aimed at providing comparable probability values, the raster outputs of these models can be simply combined with our metrics.

### Relation to terrestrial connectivity indices

Finally, we must consider how our metrics relate to other structural and functional connectivity indices (CI). Keeley et al.^40^ provide a comprehensive summary of common indices, their data requirements, and their approaches. Many of them ultimately aim to represent the fragmentation of the entire landscape. A landscape is usually considered fully connected when the entire area is a suitable habitat (i.e., consists only of suitable patches). Generally, the values of such CI are usually given as a percentage, with 0 for totally unsuitable landscapes and 1 for a contiguous patch comprising the whole landscape. In contrast, a *cP* of 1 does not mean that all patches are connected without resistance, but that, on average, each patch receives as many seeds from its neighboring patches as a populated cell would receive. Thus, values above 1 are also possible if more seeds can be exchanged between habitats.

With CI, patches are often considered a single unit (but see^39^). This is useful for animals, since they can move freely in the patch, but not for sessile organisms. For the latter, only the interface, the area of a patch that can exchange seeds with neighboring patches, is relevant. CI weighted on the total area further have a different evaluation of identical habitat configurations when the total area changes^60^. This is often the case with rivers, where, for example, the active river corridor is reduced through flood protection measures or widening after restoration. This limitation also applies to cell-based indices (e.g.^39^), which relates to the entire habitat area. Thus, with these CI, landscapes that differ in total area but have an identical habitat configuration result in different connectivity values. However, in both cases, the probability of colonization (i.e., *cP*) would be identical.

Other indices only consider a total threshold as dispersal distance, but do not consider a decreasing probability with distance. Simple distance metrics, on the other hand, such as edge-to-edge nearest neighbor distance, only take into account the nearest patch and not all patches that can contribute to colonization or the degree of reachability by seeds due to the size and shape of the interface between the patches. Also, with many CI, information at the patch level is not available (e.g.^38^). Such information, for example, the connectedness of single patches and their contribution to colonization, is vital for many applications in river restoration.

## Conclusion

The proposed metrics are particularly useful for assessing the colonization potential of riverscapes by wind-dispersed species in dynamic river systems or in open landscapes in which suitable habitats are scattered and sometimes exist only temporarily. All measures are intuitive to grasp and provide more realistic and balanced results compared to connectivity metrics. The raster-based methods can be easily supplemented by the actual occupancy, arbitrary kernels, weights (e.g., for graded habitat suitability) or even cost paths or barriers. The non-aggregated results are spatially explicit on the level of raster cells and can be directly used for further analyses and modeling. Therefore, our metrics complement existing criteria for assessing the ecological situation of rivers and provide a valuable tool for conservation and restoration planning.

### Supplementary Information

Supplementary Information.

## Data Availability

All metrics were implemented as an R package (“rconnect”) which is available through github (https://github.com/TCWagner/rconnect). An example data set is included. A previous version of this manuscript is available on researchsquare: https://doi.org/10.21203/rs.3.rs-2388009/v1.
