# Supplementary material for: A new set of metrics and framework to assess the colonization potential of riverscapes by wind-dispersed plant species
Source: Sci Rep. 2023 Nov 16;13:20097. doi: 10.1038/s41598-023-47477-y (PMC10654522; doi:10.1038/s41598-023-47477-y)
Supplement: Supplementary file 1 — Supplementary Information. [file 41598_2023_47477_MOESM1_ESM.pdf]

Thomas C. Wagner, Romy Woellner

A new set of metrics and framework to assess the colonization potential of riverscapes by wind-dispersed plant species

Appendix

Summary of major shortcomings of standard connectivity indices

Table A1: A concise summary of major shortcomings of common connectivity indices in assessing the colonization potential of wind-dispersed species in riverscapes. For the detailed characteristics and calculation on various connectivity metrics, see Keeley et al. [1]: Connectivity metrics for conservation planning and monitoring; *Biological Conservation*, *255*, 109008. https://doi.org/10.1016/j.biocon.2021.109008

**Characteristics of Connectivity Index**	**Problem associated with characteristics**
Only availability at the patch or landscape level	metric not spatially explicit, cannot be used for raster-based operations and modeling specific scenarios
Results are provided as relative values or index; ranging between 0 (unconnected) and 1 (fully connected)	No linear representation of the connectivity, e.g. 1 does not mean 5 times better connected than 0.2; no values larger than 1 possible
Each patch is considered as one homogeneous entity and patch size directly related to connectivity	Larger habitats are automatically associated with a better connectivity even if large parts of the patch do not contribute to dispersal
Only distance between patches considered	Does not account for cells further from the edge that do only contribute to a lesser extent to colonization
Connectivity related to total area of the landscape	Value for an identical spatial habitat configuration decreases if it is situated in a larger riverscape, though this does not affect the real interface and potential seed exchange between patches

Patch-to-patch values of *eC* and *eD* using the Lech Riverscape habitat situation as an example for *Chondrilla chondrilloides*.

Figure A1: Habitat configuration of the Lech Riverscape

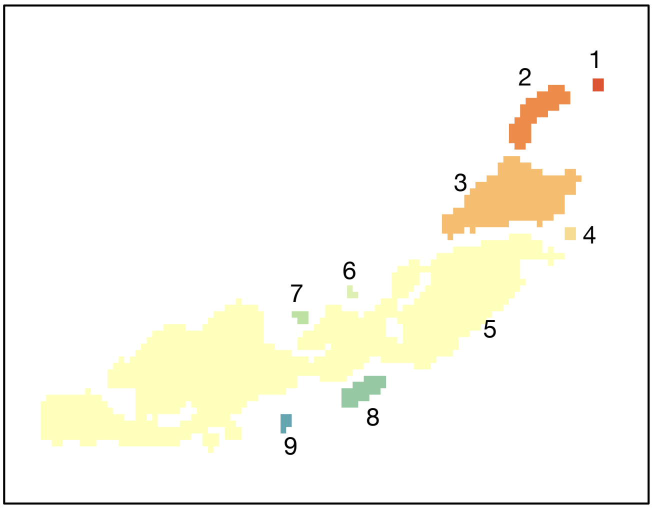

Table A2: Example of the *effective connections (eC)* matrix calculated for *Chondrilla chondrilloides* and the Lech riverscape with a threshold of 0.01 (a patch is assumed to be connected if the probability of seed reaching the patch was higher than 1%). For example, patch 1 is not connected to any other patch, while patch 5 is connected to six other patches, with the strongest connection to patch 3.

Patch	1	2	3	4	5	6	7	8	9
**1**	-	0.00	0.00	0.00	0.00	0.00	0.00	0.00	0.00
**2**	0.00	-	0.38	0.00	0.00	0.00	0.00	0.00	0.00
**3**	0.00	0.38	-	0.06	1.46	0.00	0.00	0.00	0.00
**4**	0.00	0.00	0.06	-	0.04	0.00	0.00	0.00	0.00
**5**	0.00	0.00	1.46	0.04	-	0.16	0.37	0.98	0.04
**6**	0.00	0.00	0.00	0.00	0.16	-	0.00	0.00	0.00
**7**	0.00	0.00	0.00	0.00	0.37	0.00	-	0.00	0.00
**8**	0.00	0.00	0.00	0.00	0.98	0.00	0.00	-	0.00
**9**	0.00	0.00	0.00	0.00	0.04	0.00	0.00	0.00	-

Table A3: Example of the *effective distances (eD)* matrix derived from Table 1 for *Chondrilla chondrilloides* and the Lech riverscape with a threshold of 0.01.

Patch	1	2	3	4	5	6	7	8	9
**1**	-	25.12	∞	∞	∞	∞	∞	∞	∞
**2**	∞	-	4.63	∞	∞	∞	∞	∞	∞
**3**	∞	4.63	-	13.03	0.00	∞	∞	∞	∞
**4**	∞	∞	13.03	-	14.77	∞	∞	∞	∞
**5**	∞	∞	0.00	14.77	-	8.74	4.74	0.09	15.59
**6**	∞	∞	∞	∞	8.74	-	∞	∞	∞
**7**	∞	∞	∞	∞	4.74	∞	-	∞	∞
**8**	∞	∞	∞	∞	0.09	∞	∞	-	∞
**9**	∞	∞	∞	∞	15.59	∞	∞	∞	-

Comparison of our metrics with selected distance and connectivity measures

Here, we contrast our metric's results with other commonly used distance and connectivity measures. All measures were calculated using R [2]. Edge-to-edge nearest neighbor distances (*ENN*) were determined using the *landscapemetrics* package [3], centroid-to-centroid nearest neighbor distances (*CNN*) were determine using the *spatstat* package [4]. *Class Coincidence Probability* (*CCP), Integral Index of Connectivity* (*IIC*), and *Landscape Coincidence Probability* (*LCP),* described by Pascual-Hortal and Saura [5] were calculated using the *lconnect* package [6].

While ENN and CNN were independent of the species' dispersal abilities and can be calculated on a patch-to-patch basis, the other metrics differ for identical habitat configurations according to the species' dispersal distance and are only available on the riverscape scale.

Table A4: Riverscape level comparison of *effective distance* (*eDm*), *connection capacity* (c*Cm*) and *colonization potential* (*cP*) for our three example riverscapes with common connectivity: edge-to-edge (*ENN*) and centroid-to-centroid (*CNN*) nearest neighbor distance, *Class Coincidence Probability* (*CCP), Integral Index of Connectivity* (*IIC*) and *Landscape Coincidence Probability* (*LCP)*

	**Species Independent**		***Chondrilla chondrilloides*** **SDD = 14 m**						***Myricaria germanica*** **SDD = 30 m**					
**Riverscape**	***ENN***	***CNN***	***eDm***	***cCm***	***cP***	***CCP***	***IIC***	***LCP***	***eDm***	***cCm***	***cP***	***CCP***	***IIC***	***LCP***
Lech	13.9	52.3	7.71	0.39	0.78	0.99	0.21	0.26	12.47	2.27	1.43	1.00	0.21	0.26
Wallgau	25.7	49.7	17.18	0.14	0.16	0.23	0.01	0.01	18.43	2.21	1.48	0.82	0.01	0.03
Lenggries	26.4	44.0	22.17	0.07	0.03	0.07	0.00	0.00	17.68	1.25	0.22	0.15	0.00	0.00

Table A5: Comparison of *effective distance* (*eD*) and *effective connectivity* (*eC*) with the distance measures *ENN* and *CNN* on the patch level, demonstrated for *Chondrilla chondrilloides* in the Lech riverscape

	**Species independent**		***Chondrilla chondrilloides*** SDD = 14 m	
**Patch**	***ENN***	***CNN***	***eD***	***eC***
1	10.0	59.3	25.1	0.00
2	20.0	59.3	4.6	0.38
3	10.0	58.3	0.0	1.90
4	10.0	58.3	10.5	0.11
5	14.1	29.1	0.0	3.05
6	10.0	49.3	8.7	0.16
7	10.0	29.1	4.7	0.37
8	15.8	73.4	0.1	0.98
9	25.5	55.3	15.6	0.04
**Mean**	**13.9**	**52.3**	**7.71**	**0.78**

References

[1] Keeley et al.: Connectivity metrics for conservation planning and monitoring; *Biological Conservation*, *255*, 109008. https://doi.org/10.1016/j.biocon.2021.109008

[2] R Core Team (2023). R: A Language and Environment for Statistical Computing. R Foundation for Statistical Computing, Vienna, Austria. https://www.R-project.org/.

[3] Hesselbarth, M.H.K., Sciaini, M., With, K.A., Wiegand, K., Nowosad, J. 2019. *landscapemetrics*: an open-source R tool to calculate landscape metrics. *Ecography 42*:1648-1657 (v0.0).

[4] Baddeley A, Rubak E, Turner R (2015). Spatial Point Patterns: Methodology and Applications with R. Chapman and Hall/CRC Press, London. ISBN 9781482210200

[5] Pascual-Hortal, L., & Saura, S. (2006). Comparison and development of new graph-based landscape connectivity indices: towards the priorization of habitat patches and corridors for conservation. *Landscape ecology*, *21*, 959-967.

[6] Mestre F, Silva B (2021). *lconnect*: Simple Tools to Compute Landscape Connectivity Metrics. R package version 0.1.1, https://CRAN.R-project.org/package=lconnect.
